# The Effects of Maternal Mirroring on the Development of Infant Social Expressiveness: The Case of Infant Cleft Lip

**DOI:** 10.1155/2018/5314657

**Published:** 2018-12-17

**Authors:** Lynne Murray, Laura Bozicevic, Pier Francesco Ferrari, Kyla Vaillancourt, Louise Dalton, Tim Goodacre, Bhismadev Chakrabarti, Sarah Bicknell, Peter Cooper, Alan Stein, Leonardo De Pascalis

**Affiliations:** ^1^School of Psychology and Clinical Language Sciences, University of Reading, UK; ^2^Department of Psychology, Stellenbosch University, South Africa; ^3^Department of Psychology, University of Cape Town, South Africa; ^4^Institut des Sciences, Cognitives, Marc Jeannerod CNRS, Bron, France; ^5^Spires Cleft Centre, John Radcliffe Hospital, Oxford, UK; ^6^Department of Psychiatry, University of Oxford, UK; ^7^School of Public Health, University of Witwatersrand, Johannesburg, South Africa

## Abstract

Parent-infant social interactions start early in development, with infants showing active communicative expressions by just two months. A key question is how this social capacity develops. Maternal mirroring of infant expressions is considered an important, intuitive, parenting response, but evidence is sparse in the first two months concerning the conditions under which mirroring occurs and its developmental sequelae, including in clinical samples where the infant's social expressiveness may be affected. We investigated these questions by comparing the development of mother-infant interactions between a sample where the infant had cleft lip and a normal, unaffected, comparison sample. We videotaped dyads in their homes five times from one to ten weeks and used a microanalytic coding scheme for maternal and infant behaviours, including infant social expressions, and maternal mirroring and marking responses. We also recorded maternal gaze to the infant, using eye-tracking glasses. Although infants with cleft lip did show communicative behaviours, the rate of their development was slower than in comparison infants. This group difference was mediated by a lower rate of mirroring of infant expressions by mothers of infants with cleft lip; this effect was, in turn, partly accounted for by reduced gaze to the infant's mouth, although the clarity of infant social expressions (indexed by cleft severity) and maternal self-blame regarding the cleft were also influential. Results indicate the robustness of parent-infant interactions but also their sensitivity to specific variations in interactants' appearance and behaviour. Parental mirroring appears critical in infant social development, likely supported by the mirror neuron system and underlying clinical and, possibly, cultural differences in infant behaviour. These findings suggest new avenues for clinical intervention.

## 1. Introduction

Parent-infant social interactions are foundational for later child psychological functioning [1]. These interactions start shortly after birth and develop rapidly. By two to three months, infants deploy a range of communicative expressions during face-to-face encounters, including smiles, and tongue protrusions and wide mouth openings termed “prespeech” [2, 3]. Prospective studies through the postpartum weeks have demonstrated the “functional architecture” of these interactions, whereby infant social expressions are accompanied by specific, highly organised, parental behaviours [4, 5]. Thus, parents gaze almost exclusively to their infant's face [6] and respond selectively to infant social *vs.* non-social cues by mirroring them and positively marking their occurrence with salient signals (e.g., eyebrow flashes) [2, 4, 7–10]. Such parental responses appear both intuitive and functionally important [5, 11], likely recruiting brain networks differentiating infant facial signals from other social cues [12]. Thus, the few studies focussed on the first two months indicate that parental mirroring and positive marking, and especially the former, increase the expression of infant social communication over this same period [2, 4, 7, 10] and predict later neural processing of faces [13].

Two important challenges arise from previous research: the first is to clarify the functional significance of early parental interactive behaviour by investigating it in different interactive contexts; the second is to determine the nature of infant stimuli eliciting parenting responses, that is, whether the latter depend on highly specific infant cues or are relatively robust and elicited by a broad range of infant facial expressions and configurations.

These issues are difficult to address with normal community samples, given their limited variability; however, they may be usefully elucidated in clinical conditions in which social interactions are perturbed [14]. For example, the “cute” infant facial configuration [15] may be required for intuitive parenting responses, and if infant facial stimuli are distorted by a relatively minor facial structural abnormality, such as cleft lip, alterations occur in adult gaze patterns [6, 16] and in neural responses associated with feelings of reward [12]. Whether this abnormal infant facial configuration affects key parental interactive responses like mirroring, and what mechanisms are involved, is unknown. Further, to the extent that infant cleft lip *does* influence important parental behaviours, the question arises whether this may, in turn, alter the development of infant social expressiveness. Although research shows that, by two to three months, normal levels of infant social engagement with the parent are reduced in the context of cleft lip [17–19], it is not known how these difficulties develop over the preceding weeks, or what the role might be of parental responsiveness. Given that, in this population, early interaction difficulties predict cognitive impairments in infancy and childhood [19–21], understanding how such difficulties evolve is not only of scientific interest but also of substantial clinical importance, as it could inform preventive interventions.

We addressed these issues in a prospective study of the development of infant social expressiveness and the role of parental responsiveness over the first ten postpartum weeks, comparing the development of parent-infant interactions in a normal population with those where the infant had cleft lip. We conducted systematic, naturalistic videotaped observations of interactions in the home and coded key infant social behaviours and parental mirroring, positive marking, and gaze.

Consistent with previous studies, we hypothesized that, compared to unaffected comparison infants, those with cleft lip (CLP) would not show the usual increase in social expressiveness over the first two months [4]. We also hypothesized that this relative failure to increase social expressiveness in infants with cleft lip would be predicted by reduced maternal responsiveness - particularly mirroring - to infant social cues in the CLP group.

A number of mechanisms may cause reduced maternal mirroring of infants with cleft lip. Three possible mechanisms are as follows.

- Reduced Infant Attraction Effects on Maternal Gaze. Reduced mirroring may occur because the cleft interferes with the normal parental attraction to infant cues [12, 22]. We investigated this by using eye-tracking glasses to record maternal gaze to the infant's mouth during interactions to determine whether any impact of the cleft on mirroring of social expressions could be accounted for by a reduction in gaze.
- Opacity of Infant Cues. Independently of any impact on maternal gaze, the physical alteration caused by the cleft may reduce mirroring responses by interfering with the clarity of infant social cues. Thus, despite evidence showing naïve observers' ratings of infant emotional expression in the context of craniofacial anomaly to be highly accurate [23], it has been suggested that parents of infants with clefts experience difficulty in interpreting infant behaviour during interactions [17]. We investigated this possibility, using cleft severity as a proxy for the degree of interference caused to infant facial signals.
- Maternal Mental State. Mothers have raised risk for depression in the context of infant perinatal health problems [24]. Depressive disorder can affect both maternal mirroring of infant expressions during social interactions [25, 26] and discrimination of infant emotional expressions and neural responses to infant faces [27–29]. We assessed maternal depressive symptoms to determine whether they mediated any reduction in maternal mirroring of social expressions in infants with cleft lip. Other aspects of maternal mental state might also be relevant; in particular, mothers of affected infants can experience considerable preoccupation concerning their infant's condition, with self-blame being prominent [30]. Cognitive difficulties of preoccupation, or negative rumination, concerning the mother's own role may be especially disruptive to the processing of infant cues [31]. Accordingly, we also recorded mothers' responses to the cleft itself, including feelings of self-blame. We conducted secondary analyses to see if these cognitions related to maternal mirroring in the index group.

## 2. Methods

### 2.1. Procedure

We recruited 10 infants with cleft lip, with or without cleft palate (CLP group), and 20, unaffected comparison infants. We video-recorded three minutes of mother-infant interaction at 1, 3, 5, 7, and 9 weeks, while mothers wore a mobile eye-tracker system to record their gaze. Mothers provided written informed consent. The study was approved by the NHS Research Ethics Committee (No. 11/SC/0242) and the University of Reading Ethics Committee (No. 11/45). It was conducted in accordance with the Declaration of Helsinki.

### 2.2. Measures

Infant social expressions and maternal responses were scored on a one-second time basis, using a reliable coding scheme.

#### 2.2.1. Infant Behaviour

**Figure 1:**
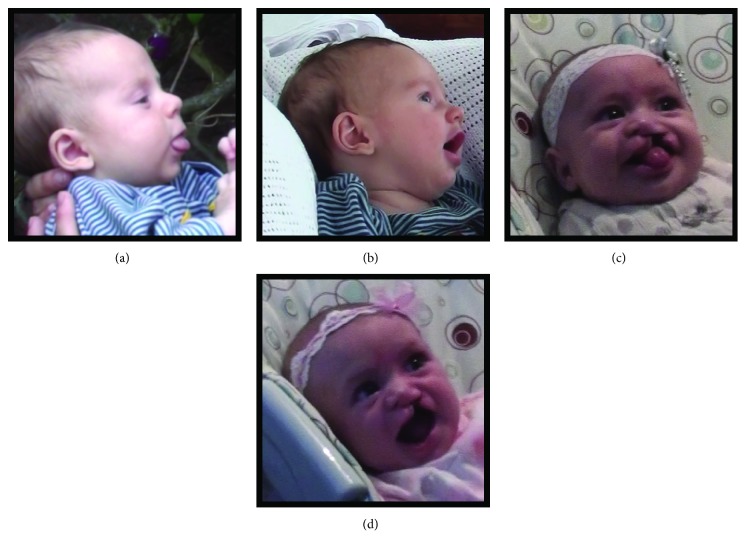
Infant prespeech mouth gestures: comparison group - (a) tongue protrusion and (b) mouth opening; CLP group - (c) tongue protrusion and (d) mouth opening.

Social facial expressions – e.g., smiles and prespeech (tongue protrusions, active wide-open shaping of mouth) (Figure 1 shows examples for both groups). These expressions have a clear structure, distinct from other, nonsocial, typically vegetative, mouth movements (e.g., low-level, continuous movements, like sucking or chewing) [3, 32].

#### 2.2.2. Maternal Behaviour

*(1) Mirroring*. Mirroring is either exact matches or else matching of the principal features of infant social behaviour with minor modification.

*(2) Positive Marking*. Positive marking is responses highlighting or “marking” infant social behaviour with smiles and “attention-attracting” cues, without mirroring.

*(3) Maternal Gaze to the Infant's Mouth*. Using the eye-tracking data, dynamic (i.e., tracking the infant's movement) areas of interest (AOI) were drawn over the infant's mouth to record the duration of maternal gaze.

#### 2.2.3. Maternal Reports (9 Weeks)

Mothers completed the Edinburgh Postnatal Depression Scale (EPDS, [33]) to assess depressive symptoms.

Mothers in the CLP group completed the Parental Appraisal of Cleft Questionnaire [30], and the self-blame factor was used.

## 3. Results

### 3.1. Sample

Maternal groups were demographically similar. Depressive symptoms were low, with both groups' mean scores being in the non-clinical range. Although infant groups differed on some measures (e.g., gestation) (Table 1), none was related to any study outcome, and they were therefore not included in further analyses (see Supplementary Materials (available here)). (One CLP group infant with later diagnosed visual impairment was excluded.)

### 3.2. Effect of Cleft Lip on Infant Social Behaviour

**Figure 2:**
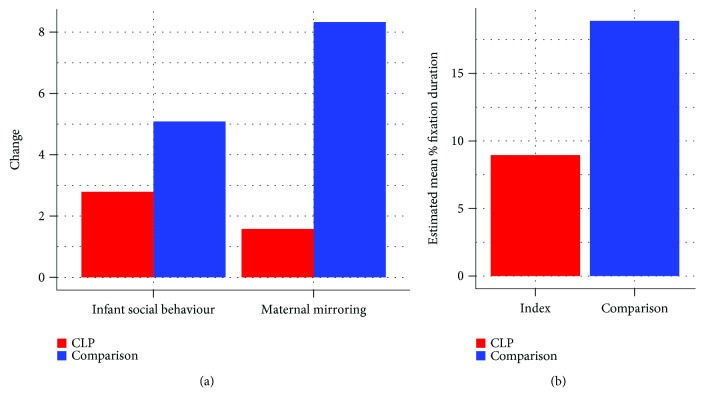
Group effects on (a) change in infant and maternal behaviour showing by how many times the rate per minute increased from the 1st to the 2nd month (e.g., for infant social behaviour, the increase over time was 2.7-fold in the CLP group vs. 5.08-fold in the comparison group) and (b) percentage of maternal gaze time to the infant's mouth.

Infant social expressiveness increased significantly over the first two months, regardless of group (Χ^2^ (1) = 508.338, *p* < .001; ERPM (estimated rate per minute) M(SE): 1st month = 1.67(0.19); 2nd month = 6.31(0.67)). The extent of increase differed, however, between CLP and comparison infants, as shown by a significant interaction between group and infant age (Χ^2^ (1) = 23.029, *p* < .001). The increase in social behaviours over time in the comparison group (from M(SE)1st month = 1.33(0.17) to 2nd month = 6.75(0.79), a five-fold increase) was significantly greater (*p* < .001) than in the CLP group (M(SE) 1st month = 2.11(0.39); 2nd month = 5.89(1.04), a 2.7-fold increase) (Figure 2(a)).

### 3.3. Maternal Responsiveness

Mirroring showed the same pattern as infant social behaviour. Thus, there was a main effect of infant age, with mirroring increasing from the first to the second month (Χ^2^ (1) = 53.123, *p* < .001; ERPM M(SE): 1st month = 0.18(0.04); 2nd month = 0.65(0.13)), and there was also a significant interaction between group and infant age (Χ^2^ (1) = 22.116, *p* < .001). In this case, only in the comparison group did mirroring increase significantly over time (M(SE) comparison - 1st month = 0.12(0.03); 2nd month = 0.98(0.20) (*p* < .001); CLP - 1st month = 0.27(0.10); 2nd month = 0.43(0.15)), with the increase being significantly greater in the comparison than in the CLP group (8.33-fold vs. 1.58-fold, respectively) (*p* < .001) (Figure 2(a)).

Importantly, these effects of infant age and group on maternal mirroring remained significant when controlling for the rate of infant social behaviours.

For positive marking, only a main effect of infant age emerged (Χ^2^ (1) = 110.332, *p* < .001; ERPM M(SE): 1st month = 0.09(0.02); 2nd month = 0.74(0.12)), with the rate increasing from the first to the second month, regardless of group. This effect remained significant when controlling for the rate of infant social behaviours.

### 3.4. The Mediating Role of Maternal Mirroring

**Figure 3:**
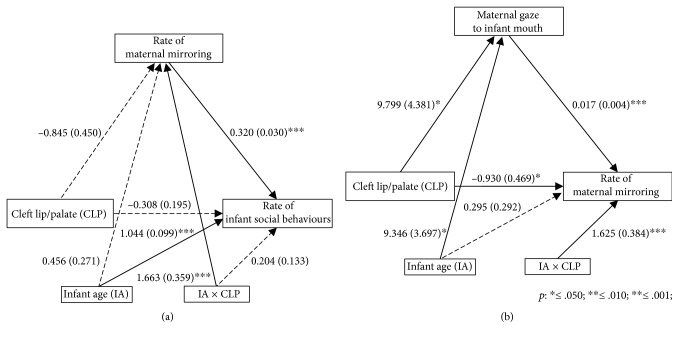
Mediation models, with unstandardised coefficients and their standard errors, showing (a) the indirect effect of the presence of a cleft lip on infant social behaviours, through maternal mirroring, and (b) the indirect effect of the presence of a cleft lip on maternal mirroring, through maternal gaze to the infant's mouth.

We then examined our key question of whether the reduced increase over time in infant social expressiveness in the CLP group compared to the control group was mediated by maternal mirroring. This was confirmed (*b* = 0.262, SE = 0.128, 95% CI = 0.011–0.513, *p* = .041) with the effect of cleft lip on infant social behaviour becoming non-significant with the inclusion of maternal mirroring in the model (see Figure 3(a)). The direct/total effect ratio (using absolute values [34]) showed that 66.06% of the effect of cleft lip on infant social behaviour was accounted for by its effect on maternal mirroring.

### 3.5. Influences on Mirroring

We next investigated possible influences on mirroring, starting with maternal gaze. There were main effects on gaze of both infant age (Χ^2^ (1) = 4.410, *p* = .036; estimated percentage M(SE): 1st month = 9.57(2.97); 2nd month = 18.27(3.17)) and group (Χ^2^ (1) = 4.830, *p* = .028; M(SE): comparison = 18.89(2.45); CLP = 8.95(3.83)). Thus, the duration of maternal gaze to the infant's mouth increased overall from the first to the second month, but it was consistently lower in CLP than in comparison group mothers (Figure 2(b)). Accordingly, we ran a second mediation model to test whether reduced gaze to the infant's mouth in CLP group mothers constituted an indirect path through which cleft lip influenced maternal mirroring. There was some evidence for this, with a significant indirect effect being found (*b* = 0.167, SE = 0.084, 95% CI = 0.001–0.332, *p* = .049). Nevertheless, the age-dependent effect of cleft lip on maternal mirroring still remained significant (see Figure 3(b)), and the direct/total effect ratio showed that the mediating effect of maternal gaze accounted for only 19.34% of the effect of cleft lip on maternal mirroring.

We then examined the influence of cleft severity on maternal mirroring, subdividing the index group in secondary analyses as follows: high severity (cleft lip and palate) vs. low severity (cleft lip only) vs. none - i.e., the comparison group. There was a significant interaction between severity and infant age on the rate of maternal mirroring (Χ^2^ (2) = 26.622, *p* < .001; ERPM M(SE): high severity cleft - 1st month = 0.33(0.14); 2nd month = 0.36(0.15); low severity cleft - 1st month = 0.16(0.10); 2nd month = 0.62(0.34); comparison - 1st month = 0.12(0.03); 2nd month = 0.98(0.20)). Neither subgroup of mothers of infants with clefts showed the extent of mirroring seen in comparison group mothers in the second month. Nevertheless, the overall lower rate of change in mirroring over time in mothers of infants with a cleft appeared to be confined to the high severity subgroup, where the change was non-significant (*p* = 0.814), as opposed to the low severity group (*p* = 0.011). These effects remained significant when controlling for the rate of infant social behaviours.

A high severity of the infant's cleft may have reduced the rate of maternal mirroring in the second month because it caused mothers to gaze away from the infant's mouth. We therefore reran the model used to investigate the mediating role of maternal gaze on mirroring, using the three-group cleft severity variable (i.e., high, low, and none). Given limited subgroup sizes, findings warrant caution. Nonetheless, a significant (*b* = −0.191, SE = 0.096, 95% CI = −0.380–(-0.002), *p* = .048) mediational role of gaze was identified for the subgroup of infants with high severity clefts, with no such effect found in the low severity subgroup. Having said this, the direct/total effect ratio showed that the mediating effect of maternal gaze accounted for only 21.87% of the effect of the high severity of the cleft on maternal mirroring.

Finally, we investigated the role of maternal mental state. There was no group difference in depression scores, precluding examination of their mediational role in explaining the effect of infant cleft lip on maternal mirroring. However, in an exploratory analysis including only the CLP group, self-blame scores emerged as significantly negatively associated with the rate of maternal mirroring (Χ^2^ (2) = 26.622, *p* < .001).

## 4. Discussion

In this novel study, capitalizing on an “experiment in nature” [23, 35] to interrogate social developmental mechanisms, we found that infants with cleft lip did not show the normal rate of increase in social behaviours over the first two months of life. This was not because the cleft prevented the performance of social expressions, since these behaviours were identified reliably and they showed an increase with age. Rather, the effect of cleft lip on infant social development was accounted for by the fact that mothers of affected infants did not mirror social expressions back to their infants to the same extent as mothers of unaffected infants. We found reduced gaze to the infant's mouth to be important in reducing mirroring in CLP group mothers, as was the severity of the cleft and maternal feelings of self blame; small sample size precluded investigation of the combined effects of these three factors.

Two key questions arise from our findings. The first concerns the mechanisms whereby maternal mirroring affects the development of infant social expressiveness. It has been suggested that mirroring is effective by virtue of its being frequent and highly contingent [36, 37]. This characterization of mirroring was not supported by our study, where, consistent with other research (e.g., [7]), it was neither frequent nor highly contingent (even in the comparison group, mirroring, although occurring highly selectively, followed only a small proportion (16%) of infant social cues [4]). Explanations for the positive effects of mirroring are therefore required that do not rely on either frequency or contingency. One hypothesis is that infants have a strong propensity to capitalise on others' mirroring [4], a propensity rooted in the preparedness of the infant brain to identify commonalities between own and others' motor patterns. In the present case, we suggest that the innate ability to generate certain active motor gestures, such as mouth openings and tongue protrusions, is complemented by a readiness to apprehend equivalence when these same gestures are observed in others ([38], p. 13). Accordingly, when the caregiver mirrors these gestures shortly following their production by the infant, the resulting instantiation of action-perception connections that are nascently present will strengthen the neural circuits involved, thereby increasing the probability of the behaviour occurring. This proposal is in line with theoretical accounts suggesting a strong canalization during development of brain circuits and related learning processes to sustain the link between infant motor facial gestures and perception of others' facial expressions [39, 40]. It is also supported by behavioural evidence, including from neonates, for the enactment and detection of imitation of others' facial gestures [41, 42], and by neurophysiological studies showing that being imitated activates areas of the STS region and inferior frontal gyrus [43], areas forming part of the *mirror neuron system* (MNS), the neural mechanism involved in self-other matching [44–46]. This mechanism has been documented early in development (e.g., [16, 47–49]), including in social contexts (e.g., [13, 50, 51]), with Rayson and colleagues finding, for example, that maternal mirroring of infant social expressions at two months predicts later infant neural processing of others' facial expressions of emotion [13].

A further possible mechanism involves the reward processing system. Thus, human and macaque studies have shown that being mirrored leads to greater reward-related responses, including self-reported liking, preferential gaze, and ventral striatal activity [52–54]. Accordingly, maternal mirroring is likely to reinforce infant communicative gestures through eliciting such reward-related activity, leading to their increase. The question of *why* being mirrored elicits reward-related activity remains open. One possibility is that it simply reduces prediction errors for encoding another's action [55]. But it is also possible that the reward system is mobilised selectively, privileging a specific subset of actions, such as prespeech gestures, that have evolved to serve communicative functions [32]. Together, these data indicate a neurofunctional architecture, whereby action-perception mechanisms in infants are sensitive and prepared to detect specific social configurations in the environment and are reinforced through behaviourally responsive, and rewarding, matching.

The second key question our findings concern is the *influences* on parental mirroring. Since the infant facial configuration of cleft lip disrupts the normal neural activation associated with feelings of attraction and motivation to interact [12], we examined parental gaze to the infant. We found that mothers did gaze less to their infant's mouth in the context of cleft lip and that this contributed to the reduction in these mothers' mirroring responses. Nevertheless, even for infants whose cleft was severe, the effect of the cleft on mirroring accounted for by reduced maternal gaze was small. This might indicate, consistent with Field and Vega-Lahr [17], that, aside from any effects on parents' overall intuitive attraction response, specific disturbances to infant facial gestures disrupt the tendency to mirror. One possibility is that fundamental biological motion dynamics [56], or kinematics, that afford perception of intentionality [57] are harder to discern in facial gestures executed in the context of cleft lip [58]. Notably, this intentional dimension of observed behaviour is key in determining imitation, even by infants [59, 60]. Another possibility, less bound up with the dynamics than with the *physical* structure of facial gestures, is that the normal signal : noise ratio of infant social expressions is reduced in cleft lip (for example, in mouth-opening gestures, the extent of change from the baseline, closed, position to its apex will be smaller in infants with a cleft than in unaffected infants).

Both suggestions might fruitfully be investigated in experimental studies of social action-perception, where critical features of infant communicative gestures are manipulated.

Finally, our study indicates the role of maternal factors in determining mirroring responses, an association already demonstrated in the context of depression [25, 26]. In the current study, consistent with previous research [18, 19], maternal depressive symptoms were not, in fact, associated with infant cleft lip. Nevertheless, mothers' beliefs about their infant's cleft, and specifically feelings of self-blame, adversely affected their interactions and reduced mirroring, suggesting that further examination of the role of maternal cognitions in infant face processing is warranted.

### 4.1. Wider Significance

#### 4.1.1. The Robustness vs. Fragility of the Parenting System and Its Influence

While we identified difficulties in the interactions of parent-infant dyads where the infant has cleft lip, it is notable that the basic structure of engagements, its “functional architecture” [4], was preserved, with key maternal responses still occurring to infant social expressions and predicting their development. The findings concerning the intactness of maternal responsiveness are consistent with experimental evidence for adults' ability to discriminate different infant emotional expressions despite facial anomalies [14, 23], and they point to the robustness of the intuitive parenting system, even under challenging conditions.

With regard to the influence of parental responses, it was notable that we found that relatively small variations in levels of mirroring had significant effects on infant social development.

While our own results are situated within the context of a clinical difficulty, the principle that modest variability in parental facial responsiveness significantly influences the development of infant social expressiveness likely has wider relevance, including the understanding of cultural variations (e.g., [61, 62]). In particular, parents' propensity to mirror certain infant behaviours, and ignore others, is likely to represent a fundamental mechanism for establishing a shared currency of communication and meaning that can then be developed and elaborated in culturally specific ways. The identification of commonalities and variations across and between cultures in the infant's linguistic environment, and their impact on auditory processing and vocal production, has received considerable research attention [63, 64]. Similar endeavour in relation to cross-cultural variation in adult-infant *facial* communication stands to add significantly to our understanding of the fundamental nature of the functional architecture of early communication.

#### 4.1.2. Clinical Implications

Our findings suggest that specific mechanisms may reduce maternal responsiveness in the context of cleft lip, including possible difficulties in recognising infant social cues, and maternal preoccupation about the infant's condition. Accordingly, interventions might usefully be directed at both these sources of difficulty. Thus, it may be helpful to support parents' awareness of their infant's communicative bids, possibly through video feedback. It may also be helpful to direct interventions at parental negative cognitions, such as self-blame, that may disturb attention to infant cues and interfere with processing their social behaviours.

### 4.2. Strengths and Limitations

#### 4.2.1. Strengths

There are almost no data on the development of social expressiveness through the early weeks, and none, to our knowledge, in clinical contexts, where sample recruitment and retention present significant challenges. Our study is therefore notable in providing evidence in this limited field, particularly since the study of clinical variations can help identify developmental processes that may remain obscure in normal populations. A further study strength is the use of a theoretically based microanalytic coding scheme to elucidate mechanisms underlying parent-infant interactions more precisely than is possible with more global measures.

#### 4.2.2. Limitations

Our clinical sample, although comparable to others including cleft lip (e.g., [58]), was small, precluding examination of the combined effects of different processes affecting maternal mirroring. These processes need further empirical examination.

## 5. Conclusions

Despite the infant facial anomaly of cleft lip, core components of the parent-infant interaction system - infant social expressions and maternal responsiveness - are retained, indicating the system's robustness. Nevertheless, interactions in the context of infant cleft lip differed from those in a normal sample, indicating that the parent-infant system is also sensitive to variation.

Maternal mirroring of infant expressions was reduced in the context of cleft lip and accounted for the slower development of affected infants' social expressiveness. The findings are consistent with evidence from neuroscience concerning the “mirror neuron system” and support theories concerning its role in early infant social development. They indicate important avenues for clinical interventions.

## Figures and Tables

**Table 1:** Sample characteristics.

			CLP *N* = 9	Comparison *N* = 20	*p*
Infants	Gestation-weeks (M(sd))		38.97(2.41)	40.79(1.60)	0.024
	Birth weight-gm. (M(sd))		2980.79(436.54)	3731.94(608.07)	0.003
	Infant gender (% male)		11.11	60.00	0.020
	Birth order (% first born)		66.67	35.00	n.s
	Infant feeding (%)	Breast	22.22	75.00	0.004
		Formula	55.56	5.00	
		Mixed	22.22	20.00	
	Cleft type (%)	Lip	33.33		
		Lip and palate	66.67		

Mothers	Maternal age (M(sd))		32.65(5.38)	33.70(2.76)	n.s
	Maternal education (% graduate)		33.33	60.00	n.s
	Maternal ethnicity (% white)		100.00	90.00	n.s
	Depression symptoms (EPDS) (M(sd))		5.71 (4.54)	4.11 (3.43)	n.s

## Data Availability

The data used to support the findings of this study are available from the last author upon request.

## Supplementary Materials

Supplementary Materials The supplementary material contains details of Procedure, Measures, and Data Analysis Plan.
