# Supplementary material for: The Effects of Maternal Mirroring on the Development of Infant Social Expressiveness: The Case of Infant Cleft Lip
Source: Neural Plast. 2018 Dec 17;2018:5314657. doi: 10.1155/2018/5314657 (PMC6311812; doi:10.1155/2018/5314657)
Supplement: Supplementary Materials — The supplementary material contains details of Procedure, Measures, and Data Analysis Plan. [file 5314657.f1.docx]

**The effects of maternal mirroring on the development of infant social expressiveness: the case of infant cleft lip**

*Participants*

The CLP group was recruited at the Spires Cleft Centre, Oxford, UK, and comprised infants with isolated cleft of the lip with and without cleft palate. Infants with additional congenital disorders were excluded. The comparison group of healthy, unaffected infants was recruited on the postnatal wards of the Royal Berkshire Hospital, to the Child Development Group Database of the School of Psychology of the University of Reading, UK.

*Procedure*

Mothers and infants were visited in the families’ homes at 1, 3, 5, 7 and 9 weeks postpartum. Assessments of mother-infant interaction took place at times when the infant was alert and contented. The infant was placed semi-reclined on their back, with the mother seated opposite; the mother was asked to interact with her infant, as she would usually do. A mirror placed behind the infant recorded the mother’s face and upper body, while one placed to the side recorded the infant’s face if they turned away from the camera. Mothers wore eye tracking glasses that were calibrated before the video-filming of the interaction started. Filming started when the mother first attempted to engage with the infant and continued for three minutes. If the infant became distressed and could not be consoled, filming stopped, and was attempted again once the infant had calmed.

*Coding*

Mother-infant interaction videos were event-coded on a one second time base, using purpose-built software for identifying associations between maternal and infant behaviour (details available from LDP).

Infant social behaviours: These were mutually exclusive, and were clearly discernible, discrete, events with definite onset. (i) ‘Pre-speech’: active movements of lips and tongue (e.g., tongue pushed into the bottom lip, moving it forward, or protruded beyond the lips), and of open mouth shaping (e.g., into an ‘O’, or pursed, as during cooing, even though unvoiced) that appear to be directed at the mother (Figure 1, main text); (ii) Smiles.

Maternal behaviours: Coded maternal behaviours occurred within two seconds of the occurrence of infant social expressions (i) Mirroring: Responses of the same valence and intensity as the infant’s behaviour that are either exact matches, or that match the principal features with some minor modification (i.e., ‘enriched’ mirroring in which some element is added (such as a vocalisation to a clear mouth opening), ‘partial’ mirroring in which some element is omitted (such as the facial expression of a cry being imitated but not the sound), or ‘modified’ mirroring in which the form is slightly changed, often in conventionalised ways (e.g., responding to an infant ‘ooo’ vocalisation with ‘goo’) (Supplementary Figure S1). (ii) Marking: Positive responses that single out and ‘mark’ an infant behaviour with ‘attention-attracting’ cues, without mirroring it, e.g., infant makes a tongue protrusion and the mother raises head and eyebrows, then nods and clearly smiles, saying ‘Is that your tongue?’ (Supplementary Figure S2).

Reliability of video interaction coding

Two trained researchers scored the videotapes. They could not be blind to infant group, but were unaware of study hypotheses. They independently coded 26 (20%) interactions, including infants of each age for both groups. Regarding infant behaviours, as shown in Figure 1, main text, infants with cleft lip do make socially expressive mouth gestures (smiles, tongue protrusions, active wide-open shaping of the mouth), similar to those in comparison infants. Consistent with previous studies (Murray et al., 2008; Montirosso et al., 2012), these were identified with high inter-rater reliability in both groups (comparison infants κ = .92; CLP infants κ = .95). Reliability for maternal mirroring and marking was similarly high (comparison mothers κ = .85; mothers of CLP infants κ = .98).

Measurement of maternal gaze and reliability

Maternal gaze was measures using Tobii Glasses 1 Eye Tracker, by Tobii Technology, Stockholm, Sweden, calibrated before recording commenced. Using the Tobii Studio Eye-tracking software (version 3.2.1.188), two trained researchers drew a dynamic (i.e. tracking the infant’s movement) area of interest (AOI) over the infant's mouth (from the bottom of the nose to the chin), during segments of footage selected to include times when the infant’s whole face was visible for > two seconds, to assess the duration of ﬁxations occurring within it. Fixations were defined as pauses of eye movement lasting > 100 milliseconds (identified using the ClearView Fixation Filter with a Velocity Threshold of 25 pixels/sample). To maximise reliability, the precision estimate provided by the Tobii Glasses 1 Eye-Tracker was used to select data, with a 50% threshold (i.e., eye-data were recorded at least 1 every 2 frames). Both researchers independently analysed a random sample of 35 videos; inter-rater correlation for the mouth AOI fixation duration values was r = 0.94.

Edinburgh Postnatal Depression Scale

Scores range from 0-30, with a cut-off of 13 identifying probable depression (Murray & Carothers, 1990)

Parental Appraisal of the Cleft Questionnaire.

This questionnaire has good reliability and validity; of the 55 items, the 6 items comprising the self-blame factor were summed and used in analyses.

Data analysis

Demographic characteristics of the two groups were compared using t-tests for independent samples, and Pearson’s chi-square tests, as appropriate.

Two-level, random intercept generalised linear mixed models, with main and interactive effects of group (CLP vs. Comparison) and infant age (1^st^ month (weeks 1 and 3) vs. 2^nd^ month (weeks 5, 7 and 9)) as predictors, were used to analyse the rate of infant and maternal behaviours (with a Poisson distribution, a log link, and duration of mother-infant interaction as offset) and the duration of maternal gaze to the infant’s mouth area (with a Gaussian distribution, and an identity link). Likelihood Ratio Tests (LRT) were used to assess the contributions of the predictors to the models. These analyses were run using R 3.3.2 (Core Team, 2016). When a significant interaction between group and infant age was found, the nlcom Stata 14 function was used (StataCorp, 2015) to investigate the effect of CLP on the change over time in the relevant variables. This was represented, using the model estimates, as the ratio of the first to the second month measurements, in order to provide a measure that was independent of variable nature and magnitude.

Using Stata 14, a generalised multilevel structural equation modelling framework was adopted to investigate mediated effects. Given limited sample size, indirect effects in mediation models were tested using the Sobel test, as opposed to bootstrap-based methods (Hayes, 2009), because of its greater conservativeness (Hayes, 2013).

Multiple comparisons were corrected using the Tukey-Kramer method. A p-value ≤0.05 was considered statistically significant.

We initially included the main effects of variables that differed by group in our analyses, but, as none was significantly related to the outcome variables, we subsequently excluded them to derive the most parsimonious models.

Leonardo De Pascalis and Lynne Murray had full access to all the data in the study, and take responsibility for the integrity of the data and the accuracy of the data analysis.

**References**

Hayes, A. F. (2009). Beyond Baron and Kenny: Statistical mediation analysis in the new millennium. *Communication Monographs, 76*, 408–420. *doi:*10.1080/03637750903310360

Hayes, A. F. (2013). *An introduction to mediation, moderation, and conditional process analysis: A regression-based approach*. New York, NY: Guilford Press.

Montirosso, R., Fedeli, C., Murray, L., Morandi, F., Brusati, R., Ghezzi, P. G., & Borgatti, R. (2012). The role of negative maternal affective states and infant temperament in early interactions between infants with cleft lip and their mothers. [*Journal of Pediatric Psychology*](https://www.researchgate.net/journal/1465-735X_Journal_of_Pediatric_Psychology)*, 37*, 241–250. *doi:* 10.1093/jpepsy/jsr089

Murray, L., & Carothers, A.D. (1990). The validation of the Edinburgh Postnatal Depression Scale on a community sample. *British Journal of Psychiatry, 157*, 288–290. *doi:* 10.1192/bjp.157.2.288

Murray, L., Hentges, F., Hill, J., Karpf, J., Mistry, B., Kreutz, M., Woodall, P., Moss, T., & Goodacre, T. (2008). The effect of cleft lip and palate and the timing of lip repair on mother-infant interactions and infant development. *Journal of Child Psychology and Psychiatry, 49*, 115–123. *doi:* 10.1111/j.1469-7610.2007.01833.x

R Core Team. (2016). R: A language and environment for statistical computing. Vienna, Austria: R Foundation for Statistical Computing. Retrieved from <https://www.R-project.org/>

StataCorp. (2015). *Stata statistical software: Release 14*. College Station, TX: StataCorp LP.

**Supplementary Figure S1. Mirroring of mouth opening**

**
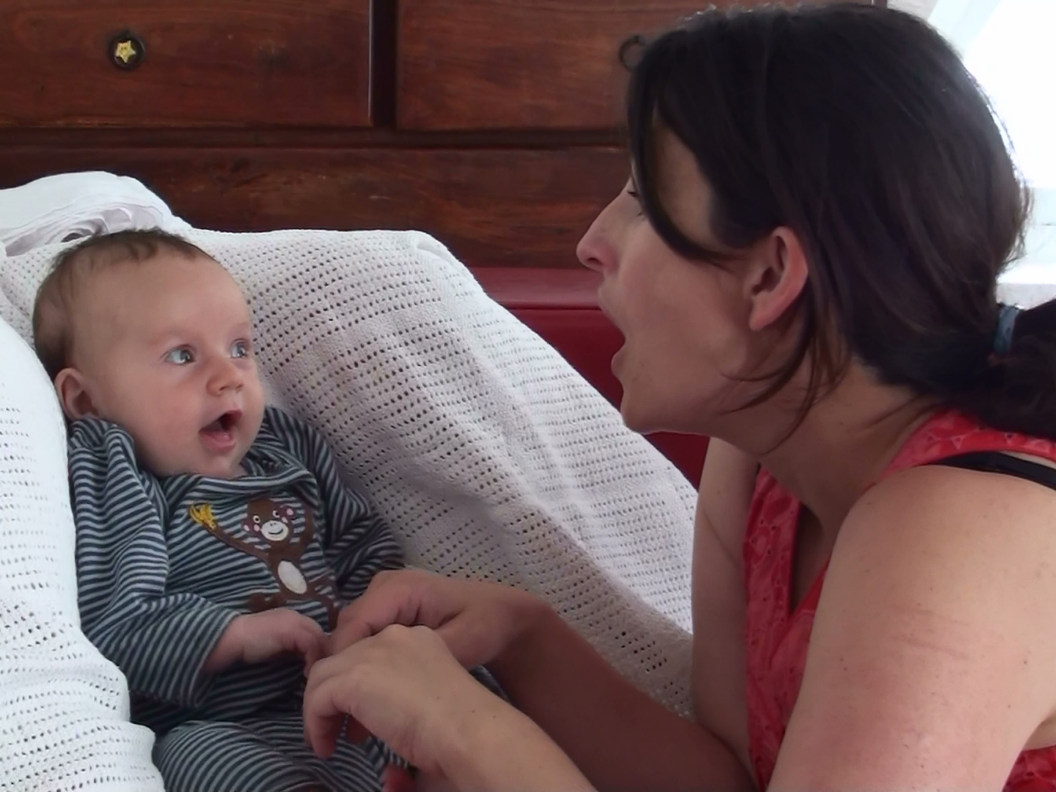
**

**Supplementary Figure S2. Marking of tongue protrusion**

**
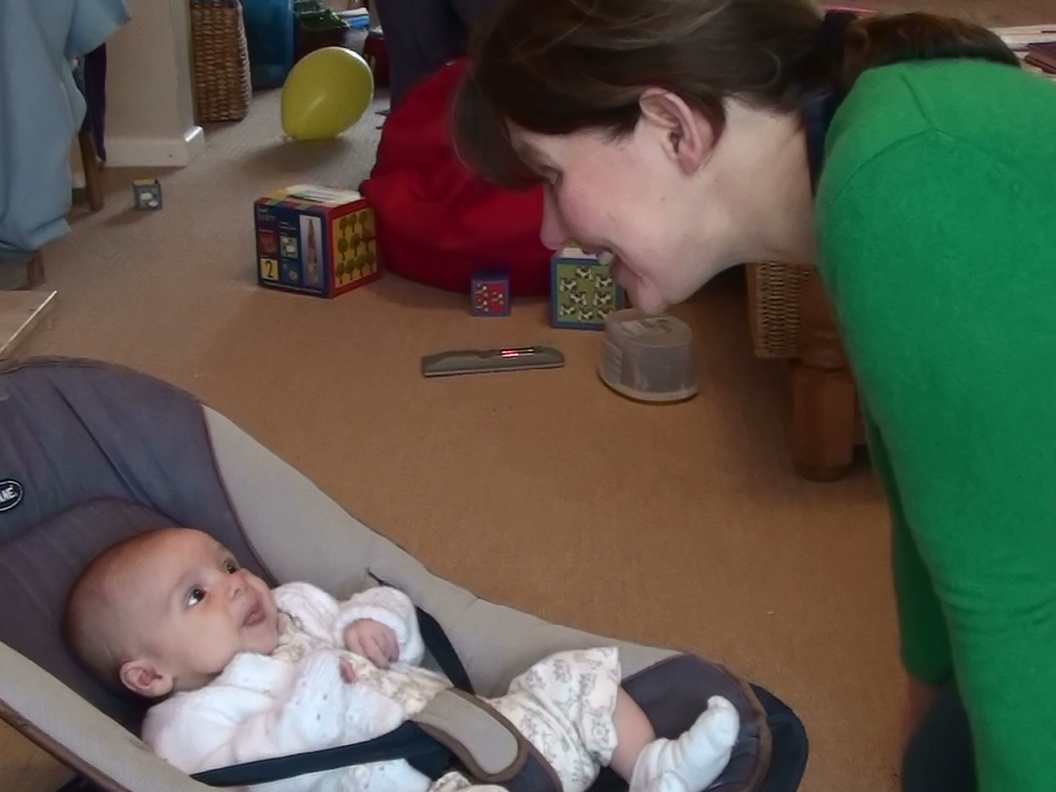
**
